# Intraocular Lens Opacification following Intracameral Injection of Recombinant Tissue Plasminogen Activator to Treat Inflammatory Membranes after Cataract Surgery

**DOI:** 10.1155/2015/975075

**Published:** 2015-03-18

**Authors:** Simon S. M. Fung, Evripidis Sykakis, Niaz M. Islam, Hadi J. Zambarakji, Ramin Khoramnia, Gerd U. Auffarth, Dipak N. Parmar

**Affiliations:** ^1^Department of Ophthalmology, Whipps Cross University Hospital, London E11 1NR, UK; ^2^Department of Ophthalmology, Queen's Hospital, Essex RM7 0AG, UK; ^3^David J Apple International Laboratory for Ocular Pathology and International Vision Correction Research Centre (IVCRC), Department of Ophthalmology, University Hospital of Heidelberg, Germany

## Abstract

*Purpose*. To report 7 cases of intraocular lens (IOL) opacification following treatment of postoperative anterior chamber fibrin with recombinant tissue plasminogen activator (rtPA) after cataract surgery. *Methods*. Retrospective case series of 7 eyes in 7 patients who developed IOL opacification after receiving rtPA for anterior chamber inflammatory membrane formation resulting from phacoemulsification cataract surgery. Three explanted IOLs were investigated with light microscopy, histochemical analysis, scanning electron microscopy, and X-ray spectrometry. *Results*. All patients underwent uncomplicated cataract surgery and posterior chamber hydrophilic IOL implantation. Anterior chamber inflammatory membranes developed between 1 and 4 weeks of surgery and were treated with intracameral rtPA. IOL opacification was noted between 4 weeks and 6 years after rtPA treatment with reduced visual acuity, and IOL exchange was carried out in 3 patients. Light microscopy evaluation revealed diffuse fine granular deposits on the anterior surface/subsurface of IOL optic that stained positive for calcium salts. Scanning electron microscopy (SEM) and energy-dispersive X-ray spectrometry (EDS) confirmed the presence of calcium and phosphate on the IOL. *Conclusions*. Intracameral rtPA, though rapidly effective in the treatment of anterior chamber inflammatory membranes following cataract surgery, may be associated with IOL opacification.

## 1. Introduction

Postoperative uveitis associated with inflammatory membrane formation occurs in less than 3% of cases after uneventful cataract surgery and intraocular lens (IOL) implantation [1]. Complications from resultant membrane formation include IOL displacement, pupillary block glaucoma, posterior capsule opacification, and side-effects from prolonged topical steroid treatment [2].

Intracameral injection of recombinant tissue plasminogen activator (rtPA), a highly potent fibrinolytic protein used for systemic thrombolysis, has been shown to successfully lyse fibrin membranes [2–5]. Reported uncommon complications of intracameral rtPA include corneal oedema, band keratopathy, anterior chamber turbidity, and hyphaema, while IOL opacification would not appear to have been previously reported [6–8].

We report 7 cases of IOL opacification subsequent to rtPA treatment for postoperative inflammatory membranes following uneventful phacoemulsification and hydrophilic acrylic one-piece IOL implantation (Rayner C-flex 570C and Superflex 620H). To the best of our knowledge, this is the first report of hydrophilic acrylic IOL opacification following the use of intracameral rtPA.

## 2. Methods

This retrospective case series included 7 eyes of 7 patients. Three patients had type 2 diabetes and treated proliferative retinopathy, and one patient presented with phacomorphic glaucoma requiring urgent cataract surgery. Two patients had medically controlled systemic hypertension but none had abnormal albumin or serum calcium levels.

All patients underwent uneventful cataract phacoemulsification and posterior chamber IOL implantation under local anaesthesia between August 2002 and September 2009. One procedure was combined with elective vitreoretinal surgery. Two percent hydroxypropylmethylcellulose (Coatel) and balanced salt solution (BSS) were used in all cases. The Rayner C-flex 570C IOL was implanted in 4 patients and the Rayner Superflex 620H was implanted in 3 patients. No remaining viscoelastic material or soft lens matter was observed at the end of all procedures. All patients received 4 hourly dexamethasone 0.1% and 6 hourly chloramphenicol eye drops postoperatively.

Inflammatory membrane formation was noted within 1 week in three patients and between 2 and 4 weeks in the remaining 4 patients. Intracameral rtPA (Actilyse) was prepared under sterile conditions using 50 mg vials of rtPA diluted with 50 mL of sterile water to create a 1 mg/mL solution. 10–50 *μ*L of this solution was injected into the anterior chamber using an insulin syringe with a 30-gauge needle. Slit lamp examination and intraocular pressure measurement were performed 2 and 24 hours after rtPA treatment. The frequency of dexamethasone 0.1% was increased to be hourly by day, together with chloramphenicol 6 hourly and cyclopentolate 1% 8 hourly. Further reviews were scheduled at weeks 1 and 3 and at 6 months after treatment.

Intraocular lens exchange was performed in 3 patients. Opacified IOLs were viscodissected with sodium hyaluronate (Healon), with care taken to avoid manipulation of the opacified portion. One patient required bisection of the IOL and anterior vitrectomy due to severe adhesion between the IOL and lens capsule. Explanted IOLs were placed in a sterile container with neutral buffered formalin 10% before they were sent for laboratory analysis.

### 2.1. Laboratory Analyses

Two explanted IOLs were sent to the Laboratories for Ophthalmic Devices Research, Sullivan's Island, South Carolina, USA, for light microscopy and histochemical analysis. Another IOL was sent for light microscopy, scanning electron microscopy, and X-ray spectrometry at the International Vision Correction Research Centre, Department of Ophthalmology, University of Heidelberg, Germany.

Detailed techniques for preparation and staining of explanted IOLs for calcium have been described elsewhere [9]. Briefly, IOLs were photographed under a light microscope and were subsequently treated with special stains for calcium (von Kossa 0.5% and alizarin red 1%). The IOLs were reexamined and photographs were again taken. Full-thickness sections were made through the opacified portion and the IOLs were restained for cross-sectional examination. Scanning electron microscopy (SEM) and energy-dispersive X-ray spectrometry (EDS) for elemental analysis were also separately performed.

## 3. Results

Table 1 summarises the preoperative comorbidities, procedures performed, and subsequent treatment for the 7 patients. Inflammatory membranes developed between 1 and 4 weeks postoperatively, and resolution of inflammatory membrane occurred within 24 hours after intracameral rtPA in all cases. Patients presented with IOL opacification between 4 weeks and 8 months after treatment. IOL exchange was carried out in 3 patients with a mean final BCVA of 0.20 logMAR. Three other patients declined IOL exchange and 1 patient was deemed unsuitable for further surgery due to poor visual potential secondary to neovascular glaucoma.

**Figure 1:**
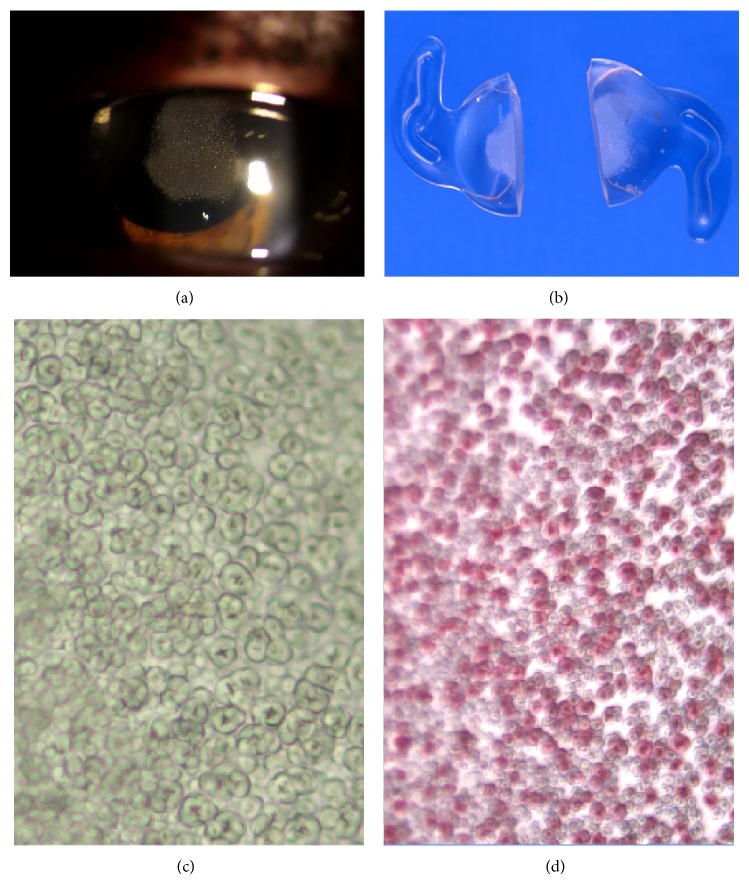
(a)–(d) Patient 4: (a) slit lamp photograph showing refractile fine granular opacities of the anterior IOL surface; (b) explanted bisected IOL; (c) high power photomicrograph (unstained) showing granular infiltration beneath anterior IOL surface; (d) positive staining with alizarin red (OM ×100). (IOL: intraocular lens; OM: original magnification).

Macroscopic and microscopic findings on all explanted IOLs were highly comparable. On macroscopic examination, fine granular whitish opacities were observed in the central part of all explanted IOL optics (Figure 1). Light microscopy revealed diffuse fine granular deposits on as well as below the anterior surface of the nonencapsulated optic. These deposits stained positively with von Kossa and alizarin red and were linearly distributed parallel to the anterior IOL surface, diminishing towards the periphery and posteriorly. Granular material was not observed on the posterior region of the bisected optic.

**Figure 2:**
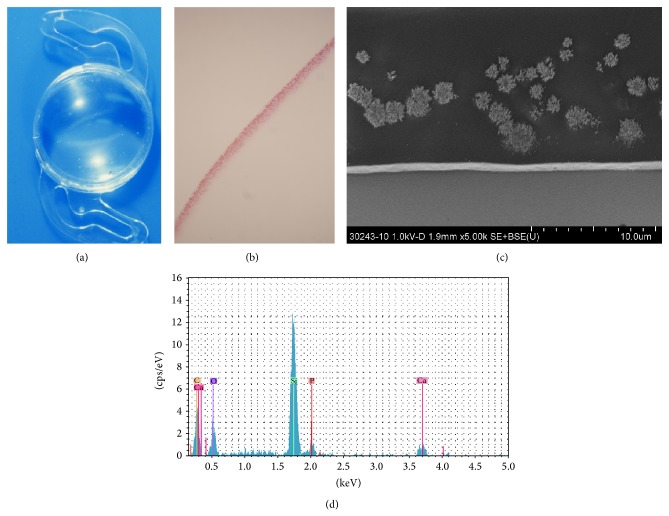
(a)–(d) Patient 2: (a) explanted IOL shows a band shaped configuration of fine white granular material in the central part of the optic. (b) Cross section through IOL shows positive granular staining with alizarin red. The granules are arranged linearly and diminish towards the periphery of the IOL optic (OM ×40). (c) The deposits are distributed below the surface of the IOL (OM ×5000, scanning electron microscopy). (d) Energy-dispersive X-ray spectrometry confirms the presence of calcium (Ca) and phosphate (P) in the deposits. Note that the spike for silicon (Si) is an artefact caused by a silicon wafer which was used for the analysis. (IOL: intraocular lens; OM: original magnification).

Cross-sectional SEM of opacified IOL showed crystalline deposits distributed close to the surface of the IOL (Figure 2). Analysis with EDS confirmed the presence of calcium and phosphate (Figure 2(d)) within the compound.

## 4. Discussion

IOL opacification is an infrequent complication after uneventful cataract surgery, resulting in symptoms of visual loss or glare, necessitating IOL exchange in some cases. To our knowledge, this is the first report of IOL opacification due to calcification following the use of rtPA for postoperative fibrinous membranes after cataract surgery.

A number of cases of IOL opacification have previously been reported; almost all were involving hydrophilic acrylic IOLs from various manufacturers [9–15]. It is noteworthy that Lee et al. reported 2 cases of surface calcification of hydrophilic acrylic Rayner C-flex 570C implants related to inflammatory membrane formation after combined vitrectomy and cataract surgery [15]. Similar to their report, we found localised calcification on the anterior and subsurface within the capsulorhexis area on both the C-flex and the Superflex IOLs. Such calcification is independent of the manufacturing and packaging process and is thus classified as secondary calcification relating to environmental causes [16].

IOL calcification has been associated with systemic disease, intraocular surgery, inflammation, or drug administration [5, 9, 16–18]. Breakdown of blood-aqueous barrier (BAB) and disruptions to aqueous calcium homeostasis are thought to lead to dystrophic calcium-phosphate precipitation on the IOL, although the precise mechanism remains unclear. Recently Ahad et al. reported a case series of 15 patients with hydrophilic acrylic IOL opacification following Descemet stripping automated endothelial keratoplasty (DSAEK), in which they identified air injection for DSAEK graft rebubbling as a significant risk factor [19]. Another recent study described 5 cases of hydrophilic acrylic IOL opacification following uneventful DSAEK [20]. Analysis of the opacified IOLs demonstrated findings identical to those observed in our study, using similar methodology.

In our series, all cases had postoperative inflammatory membranes that were successfully treated with rtPA. In our unit, rtPA was more commonly used in phakic patients with fibrinous uveitis. We are not aware of any other pseudophakic patients who had received intracameral rtPA injections. Thus it would be suggested that intracameral rtPA injection is an absolute risk factor for IOL opacification. In response to our findings, we stopped further usage of intracameral rtPA in cases of postoperative inflammatory membrane formation, and we have not observed any cases of IOL opacification since. These cases were also reported to the Medicines & Healthcare Products Regulatory Agency in the United Kingdom.

We hypothesise that rtPA contributes to IOL opacification through its action on aqueous calcium-phosphate homeostasis and possible disruption of the blood-aqueous barrier (BAB). Intracameral rtPA lyses fibrinous inflammatory membranes and in doing so releases sequestered calcium from the fibrinous matrix. It also introduces phosphate ions contained in its buffer solution, which may potentiate abnormal calcium precipitation. A case report of band keratopathy formation after rtPA injection lends support to our hypothesis [5]. Recent laboratory model has also shown that calcification occurred when the IOL was exposed to a supersaturated solution of calcium and phosphate ions, and the reaction is initiated within the IOL towards the anterior IOL surface due to ionic diffusion [21]. This coincides with our observation and may explain the preferential calcification on the anterior subsurface/surface of the hydrophilic IOL.

Similar to air injection in DSAEK graft rebubbling [19], rtPA injection may also lead to further disruption of the BAB. This may be due to the induction of nonplasminogen intracellular signalling pathways [22, 23] or the mechanical effect of the injection itself. Further investigations on the effect of rtPA on BAB are certainly warranted.

We have considered alternative hypotheses for our findings. Although intraocular inflammation could be solely responsible for IOL opacification, it fails to explain the strong association between IOL opacification and the use of rtPA. The dosages of rtPA used in our patients were within the range reported by previous authors (3–25 *μ*g) in all except 2 cases [2–4, 24]. It is plausible that the higher doses in the 2 cases may have exacerbated the extent of IOL opacification, although this has not been formally quantified.

Another possible cause could be the IOLs we used in these patients. Cases of IOL opacification occurred during a period when our unit exclusively used Rayner IOLs. C-flex and Superflex differ in the size of the optic (5.75 mm and 6.25 mm in diameter, resp.) and the range of diopter power (+8.0 to +34.0D and −10.0 to +22.0D, resp.), but they are both hydrophilic acrylic one-piece monofocal IOLs. However, there was no evidence of any manufacturing defects on the explanted IOLs in our histopathological analysis. Opacification of hydrophilic acrylic lenses from other manufacturers has also been previously reported. Therefore, we do not believe our findings are IOL-specific phenomenon.

This study highlights that intracameral injection of rtPA for the treatment of postoperative inflammatory membranes could lead to IOL opacification requiring IOL exchange. The limitations of this study were its retrospective nature and the small number of cases involved. The intraocular effects of rtPA may be more extensive than previously thought, and further laboratory investigations on this are needed. Caution should be exercised in using rtPA to treat inflammatory membranes following intraocular surgery, especially in the presence of hydrophilic IOLs.

## Figures and Tables

**Table 1:** Characteristics of patients with IOL opacification following intracameral rtPA treatment after uncomplicated cataract surgery.

Case	Eye	Ocular comorbidity	Additional procedures	IOL type	Presenting BCVA	Time from surgery to rtPA	rtPA dose (*μ*g)	Time of IOL opacification after rtPA	After rtPA BCVA	IOL exchange	Final BCVA
1	OS	—	—	C-flex 570C	0.00	1 week	25	3 months	0.00	Yes	0.00
2	OD	—	—	C-flex 570C	0.40	4 weeks	25	4 months	0.00	Yes	0.30
3	OD	—	—	Superflex 620H	0.10	2 weeks	25	8 months	0.30	Yes	0.10
4	OS	Phacomorphic glaucoma	—	C-flex 570C	0.80	2 weeks	10	6 weeks	0.90	Declined	0.60
5	OD	Treated PDR, previous vitrectomy and delamination	—	Superflex 620H	0.48	1 week	20	17 months	0.30	Declined	0.60
6	OD	Treated PDR, persistent DMO	Combined with IVTA	C-flex 570C	1.00	2 weeks	50	4 weeks	0.78	Unsuitable	2.00
7	OS	PDR, vitreous haemorrhage	Combined with PPV, delamination and laser	Superflex 620H	1.00	1 week	40	12 months	0.40	Declined	0.50

PDR: proliferative diabetic retinopathy, DMO: diabetic macular oedema, IOL: intraocular lens, BCVA: best corrected visual acuity (measured in logMAR), and *μ*g: microgram.
